# *Escherichia coli* O157:H7 strains harbor at least three distinct sequence types of Shiga toxin 2a-converting phages

**DOI:** 10.1186/s12864-015-1934-1

**Published:** 2015-09-29

**Authors:** Shuang Yin, Brigida Rusconi, Fatemeh Sanjar, Kakolie Goswami, Lingzi Xiaoli, Mark Eppinger, Edward G. Dudley

**Affiliations:** Department of Food Science, The Pennsylvania State University, University Park, PA 16802 USA; Center of Molecular Immunology and Infectious Disease, The Pennsylvania State University, University Park, PA 16802 USA; Department of Biology and South Texas Center for Emerging Infectious Diseases, University of Texas at San Antonio, San Antonio, TX 78249 USA; 427 Food Science Building, The Pennsylvania State University, University Park, PA 16802 USA

**Keywords:** *Escherichia coli* O157:H7, Shiga toxin, Bacteriophage, Genomics

## Abstract

**Background:**

Shiga toxin-producing *Escherichia coli* O157:H7 is a foodborne pathogen that causes severe human diseases including hemolytic uremic syndrome (HUS). The virulence factor that mediates HUS, Shiga toxin (Stx), is encoded within the genome of a lambdoid prophage. Although draft sequences are publicly available for a large number of *E. coli* O157:H7 strains, the high sequence similarity of *stx*-converting bacteriophages with other lambdoid prophages poses challenges to accurately assess the organization and plasticity among *stx*-converting phages due to assembly difficulties.

**Methods:**

To further explore genome plasticity of stx-converting prophages, we enriched phage DNA from 45 ciprofloxacin-induced cultures for subsequent 454 pyrosequencing to facilitate assembly of the complete phage genomes. In total, 22 stx2a-converting phage genomes were closed.

**Results:**

Comparison of the genomes distinguished nine distinct phage sequence types (PSTs) delineated by variation in obtained sequences, such as single nucleotide polymorphisms (SNPs) and insertion sequence element prevalence and location. These nine PSTs formed three distinct clusters, designated as PST1, PST2 and PST3. The PST2 cluster, identified in two clade 8 strains, was related to *stx2a*-converting phages previously identified in non-O157 Shiga-toxin producing *E. coli* (STEC) strains associated with a high incidence of HUS. The PST1 cluster contained phages related to those from *E. coli* O157:H7 strain Sakai (lineage I, clade 1), and PST3 contained a single phage that was distinct from the rest but most related to the phage from *E. coli* O157:H7 strain EC4115 (lineage I/II, clade 8). Five strains carried identical *stx2a*-converting phages (PST1-1) integrated at the same chromosomal locus, but these strains produced different levels of Stx2.

**Conclusion:**

The *stx2a*-converting phages of *E. coli* O157:H7 can be categorized into at least three phage types. Diversification within a phage type is mainly driven by IS*629* and by a small number of SNPs. Polymorphisms between phage genomes may help explain differences in Stx2a production between strains, however our data indicates that genes encoded external to the phage affect toxin production as well.

**Electronic supplementary material:**

The online version of this article (doi:10.1186/s12864-015-1934-1) contains supplementary material, which is available to authorized users.

## Background

Shiga toxin-producing *Escherichia coli* (STEC) O157:H7 is a food- and waterborne human pathogen that causes diarrhea, hemorrhagic colitis (HC), or in more severe cases hemolytic uremic syndrome (HUS) [1, 2]. Cattle are the primary reservoir of this pathogen and may be asymptomatically colonized [3]. Due to the serious disease and its low-infectious dose [4], *E. coli* O157:H7 is considered an adulterant in non-intact raw beef products in the United States. Shiga toxin (Stx) is one of the major virulence factors involved in *E. coli* O157:H7 pathogenesis [5]; based on immunoreactivity, toxins are classified as either Stx1 or Stx2 [6]. DNA sequence diversity further divides Stx2 into 7 allelic types (Stx2a through Stx2g) [7] with Stx2a and Stx2c most frequently associated with HUS [8, 9]. During the stepwise evolution of *E. coli* O157:H7 from a common *E. coli* O55:H7 ancestor, genes encoding Stx1 and Stx2 were acquired horizontally by phage transduction [10]. In this established model, the *stx2*-converting phage was acquired before the antigenic shift from O55 to O157, followed by *stx1*-converting phage acquisition. Follow-up studies refined this model [11, 12] and most recently, Lacher *et al.* [13] proposed a model taking into consideration acquisition and loss of *stx1*-, *stx2a*-, and *stx2c*-converting phage, and additional mutations that define *E. coli* O157:H7 sub-lineages. Genetic characterization together with clinical data has identified that two groups, designated clade 6 and clade 8, are more commonly associated with severe disease than are isolates from other clades [14, 15].

The lambdoid *stx*-converting phages are temperate, exhibiting both lytic and lysogenic life cycles [16]. The lysogen-lytic switch is directed by several regulatory proteins including *cI*, *cII*, *cIII*, *cro*, N, and Q, the latter of which activates transcription from the late promoter, *p*_R’_. The genes encoding Stx2 are positioned downstream of *p*_R’_ [17], and DNA damaging agents that activate lytic replication also increase toxin production [18]. Previous genomic analyses [19, 20] demonstrated that DNA sequence variation between *stx2*-converting phages occurs particularly within the regulatory regions, leading to the hypothesis that differences in the kinetics and abundance of prophage induction may be responsible for the variations in Stx2 production observed between different *E. coli* O157:H7 strains [21, 22]. This is supported by the observation that the *E. coli* O157:H7 *Q* allele designated Q_933_ is a marker of high Stx2a producing strains [23], although this has not been seen in other isolate collections [24]. Other factors impacting toxin production include the presence of insertion sequences (IS) [24–26] within the phage genomes, and *cI* repressors encoded within other prophage [27].

In previous publications we characterized 52 *E. coli* O157:H7 isolates from patients in Pennsylvania [28] and sequenced the genome of three isolates [29]. While the whole-genome sequence (WGS) data provided valuable information concerning strain diversity, the *stx*-converting phage genomes, critical for understanding toxin production, could not be fully assembled. Factors contributing to this include the short read technology used, and the high sequence identity with other lambdoid phages; e.g. 13 out of 18 prophages in *E. coli* O157:H7 Sakai are classified as lambdoid [30]. The objectives of the present work were to isolate and sequence *stx2a*-converting phages from a diverse set of *E. coli* O157:H7 strains, and to compare these phage genomes to those previously sequenced; the identification of identical phage within different *E. coli* O157:H7 hosts also allowed us to test whether factors encoded outside of the phage genome impact Stx2 production.

## Results

### Phage genome assemblies

To enrich phage DNA (phDNA), *E. coli* O157:H7 strains were induced by ciprofloxacin and total phDNA was isolated. For 22 of the 45 strains, sufficient dsDNA (≥200 ng) was obtained for 454 sequencing, and assembly resulted in complete *stx2a*-converting phage genomes ranging in size from 61,292 to 65,334 bp (Table 1, Additional file 1: Table S1). In addition, two 15,957 bp phage genomes unrelated to *stx2a*-converting phage and inserted at *yicC*, yielded enough read coverage for complete phage assembly.

**Table 1:** Genome size, genomic insertion site, IS*629* copy number, and host strain(s) for the nine Phage Sequence Types (PSTs) defined in this study

PST	Genome Size (bp)	Insertion site	IS629 copy number	Host Strain(s)
1-1	62708	*wrbA*	1	PA4, PA5, PA11, PA16, PA18, PA21, PA29, PA30, PA32, PA33, PA42, PA44, PA50
1-2	61388	*wrbA*	0	PA12, PA51
1-3	64021	*wrbA*	2	PA27
1-4	64021	*wrbA*	2	PA36
1-5	65334	*wrbA*	3	PA45
1-6	65334	*wrbA*	3	PA52
2-1	63569	tRNA(*argW*)	1	PA2
2-2	63584	tRNA(*argW*)	1	PA8
3-1	61292	tRNA(*argW*)	2	PA28

### Twenty-two *stx2a*-converting phage genomes form three genetic clusters

**Fig. 1:**
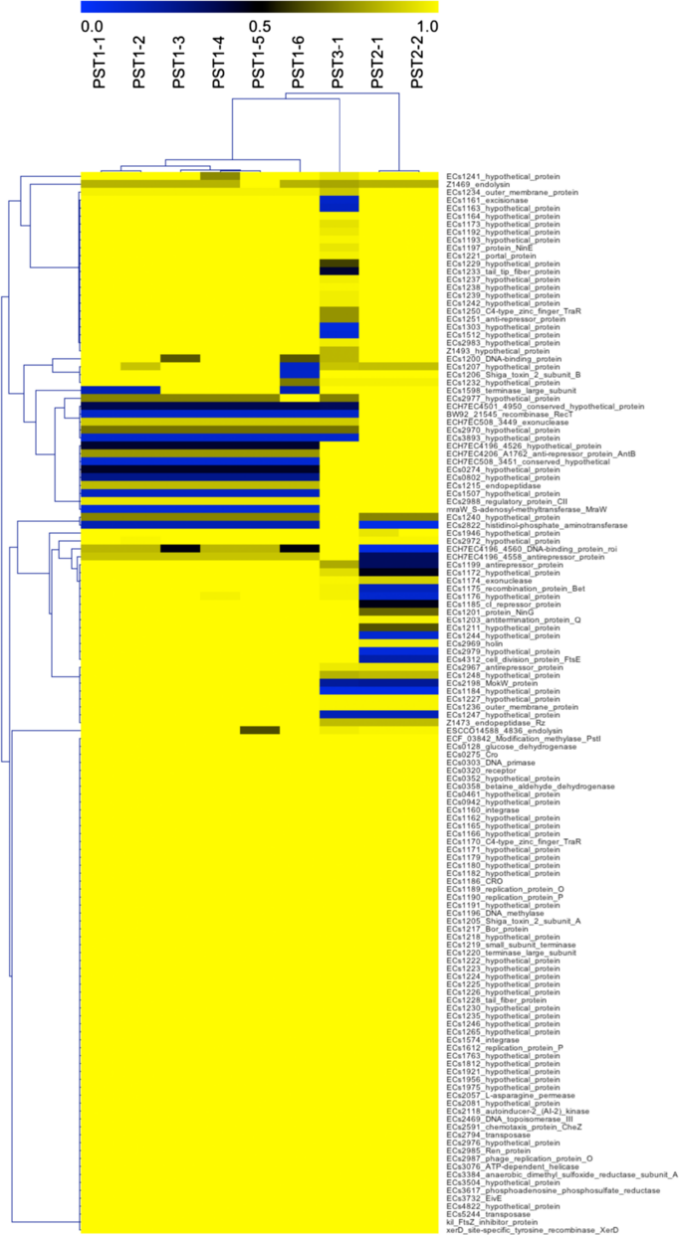
Nine phage sequence types (PSTs) form three clusters when compared at the proteomic level. *De novo* gene prediction with prodigal was used to have consistent ORF prediction prior to tblastn analysis. Resulting LS-BSR values were clustered by average linkage hierarchical clustering resulting in three distinct main phage clusters (PST-1, PST-2, PST-3). Five proteins were highly conserved in PST2, 4 in PST3 and 6 in PST1, and almost 50 % of proteins are conserved in all phages. The LS-BSR for each ORF identifies unique (*Blue* = 0), divergent (*Black* = 0.5) and common proteins (*Yellow* = 1.0) [87]

**Fig. 2:**
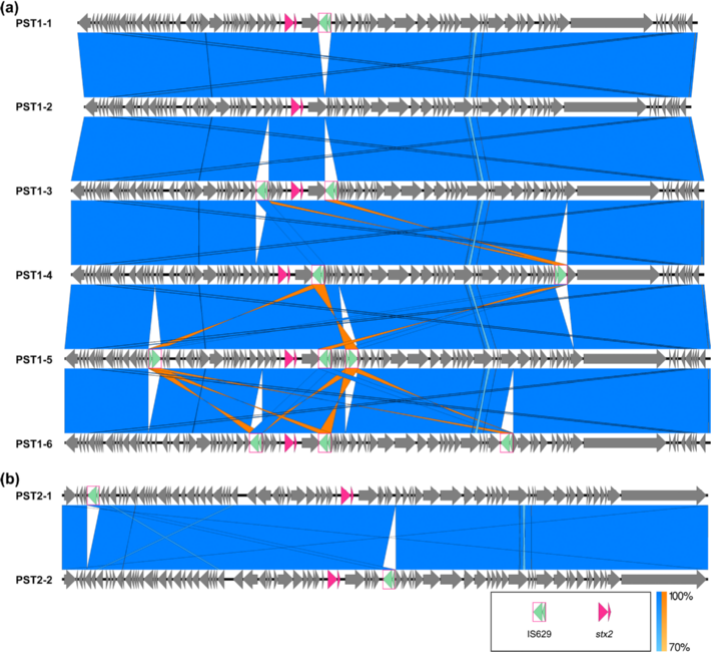
IS*629* is responsible for most diversity observed within (**a**) PST1 and (**b**) PST2 clusters. BLAST comparisons of phage genomes were visualized using Easyfig [77]. Blue shaded regions connect homologous sequences and orange shaded regions connect reverse complemented homologous sequences. Arrows represent ORFs. Dark pink and green arrows designate the location of *stx2a* and IS*629*, respectively

Predicted phage proteomes were compared with Large Scale Blast Score Ratio (LS-BSR) [31]. Applying hierarchical average linkage clustering algorithm, which is part of MeV v.4.8 [32], on this proteomic matrix (Pearson Correlation) [33] identified three distinct phage clusters, designated as Phage Sequence type 1 (PST1), PST2 and PST3 (Fig. 1). These three clusters were also apparent within a whole genome alignment (Additional file 2: Figure S1). The 19 genomes classified as PST1 were further divided into six subtypes (PST1-1 to PST1-6) by assessing IS*629* copy number and insertion sites (Fig. 2a). PST1-1 contained 13 phage genomes that each have IS*629* inserted at the same position (nucleotides 24,350–25,659), splitting a predicted phage protein into two predicted proteins (as an example, those with protein_id AKI86024 and AKI86027 in the PA4 phage GenBank entry, KP682372). This insertion sequence is located downstream of the *stx2* gene. Genome sequences of the 62 kb PST1-1 phage differed at most by five SNPs, and these SNP level differences were observed within the predicted O_R2_ operator, portal protein, tail fiber, and several hypothetical proteins (Additional file 3: Table S2). All PST1-1 isolates carry the *stx2a*-converting prophage found in the prototypical strain *E. coli* O157:H7 Sakai [30]. The two phages in PST1-2 were 100 % identical on the nucleotide level, and PSTs 1–3, −4, −5, and −6 each represent single phage found in strains PA27, PA36, PA45, and PA52, respectively.

We found that all PST1 cluster phages inserted within *wrbA*, which encodes a TrpR binding protein, while both the PST2 and PST3 cluster phages were inserted within the tRNA *argW* (Table 1). The *int* sequences were identical for the PST2 and PST3 cluster phages. However, these were clearly of different evolutionary origin or have substantially diverged from *int* encoded within PST1 cluster phage, as neither the DNA nor the deduced amino acid sequences could be aligned using BLAST [34]. A correlation between *int* sequences and insertion site has been noted before [35].

### IS*629* insertion and position is a main driver of genetic variation observed within PST1 and PST2 clusters

Genomes from the PST1 phage cluster ranged in size from 61,388 to 65,334 bp (Table 1). IS*629* copy numbers, ranging from 0 (PST1-2) to 3 (PST1-5 and PST1-6), accounted for most of this difference. The insertion site and orientation of IS*629* varied as well (Fig. 2a and Additional file 4: Table S3). For instance, for PSTs 1–3 and 1–6, the transposon inserted at position 25,663, interrupting the gene for a putative DNA-binding protein Roi. IS*629* also inserted at position 28,390 in PST1-5, interrupting a putative lysozyme, and at position 2456 in PST2-1, interrupting a putative adenine DNA methyltransferase. In PST2-2, the insertion was found at position 31,540 within an intergenic region (Fig. 2b and Additional file 4: Table S3).

### Phage early regulatory and replication regions differed among the three clusters

**Fig. 3:**
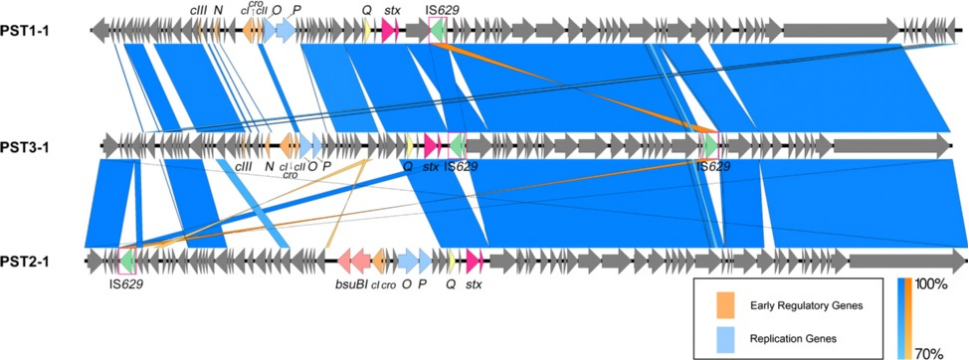
The three PST clusters differ mainly in the early regulatory and replication regions. BLAST comparisons of phage genomes were visualized using Easyfig [77]. Blue shaded regions connect homologous sequences and orange shaded regions connect reverse complemented homologous sequences. Arrows represent ORFs. Light orange, light blue and light yellow arrows represent early regulatory genes, replication genes and antiterminator *Q*, respectively. Light pink arrows represent *bsuBI* restriction/modification system. Dark pink and green arrows designate the location of *stx2* and IS*629*, respectively

We next performed a global alignment of representative phage genomes from PST1, PST2, and PST3 (Fig. 3). Most differences were observed within regions predicted to encode the phages’ early regulatory and replication genes. For example, *cI* (annotated as protein_id AKI86003 in the PST1-1 phage from PA4, AKI85932 in the PST2-1 phage from PA2, and AKI86748 in the PST3-1 phage from PA28) and *cro* (protein_id AKI86003 in the PA4 phage, AKI85933 in PA2, and AKI86749 in PA28) only shared 9.8–49.8 % amino acid sequence identity over the full gene length among the three clusters (Additional file 5: Table S4). Homologs that were distantly related by amino acid identity could still be identified by their shared patterns defined in the Clusters of Orthologous Groups database [36]. Predicted operator sequences [37, 38] were identified between *cI* and *cro* (O_R1_, O_R2_, and O_R3_) and between *cI* and *N* (O_L1_, O_L2_, and O_L3_) in genomes of the PST1 and PST3 cluster (Additional file 6: Table S5). We could not identify putative operator sequences or homologs to the early regulatory genes *cIII* and *cII* in the PST2 cluster, but instead a type II restriction/modification enzyme system (*bsuBI*) was found at the corresponding region.

The sequence divergence within predicted early regulatory and replication genes is contrasted by the conservation of predicted late and structural genes. For instance, one of the late genes encoding a putative lysozyme (protein_id AKI86033 in PA4, AKI85950 in PA2, and AKI86778 in PA28), shared 95–98 % amino acid sequence identity over the full gene length among the 3 clusters (Additional file 5: Table S4).

### The PST2 cluster is more closely related to the *stx2a*-converting phage of *E. coli* O104:H4 2011C-3493 rather than to PST1 and PST3

**Fig. 4:**
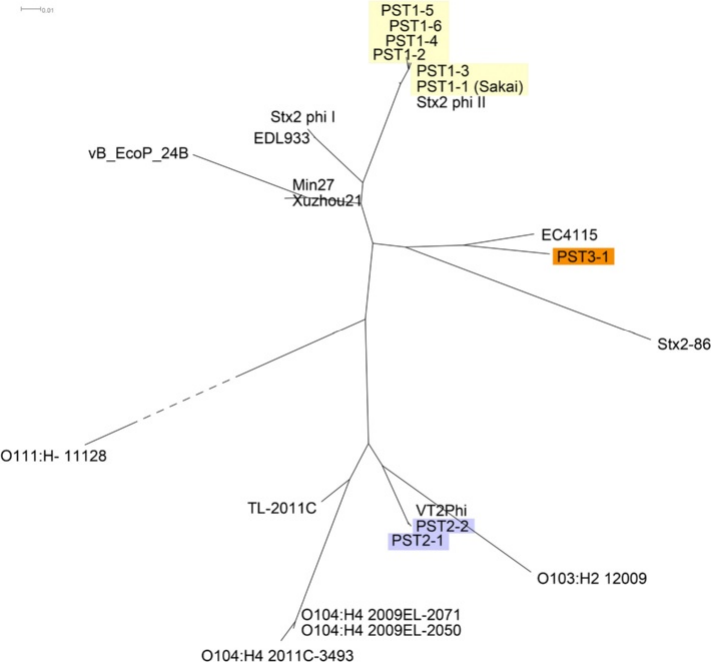
Phages of PST2 are related to those from strains associated with high HUS rates. Host strain names or phage names are indicated. Phage genomes were cut into 500 bp fragments. The “Core genome” was defined as fragments present in >2 phage genomes at > 85 % identity using Panseq [39]. The presence or absence of core genome fragments in each phage genome was used for clustering. Uncorrected P-distance was used to build a neighbor-joining tree using SplitsTree 4 [80]. PST1, PST2, and PST3 clusters are highlighted by yellow, light purple and orange shading, respectively

Phage genomes were extracted from 10 closed bacterial genomes deposited in GenBank (Additional file 7: Table S6), and we compared these to the nine PSTs established in this study and to seven other representative *stx2a*-converting phages from the *E. coli* O157:H7 lineage and non-O157 serotypes. We identified sequence fragments present in at least 3 of the 24 genomes using Panseq [39], and their distribution, presence or absence, in each phage genome was used to infer a neighbor-joining tree [40] using SplitsTree 4 (Fig. 4). The PSTs within a cluster were located on the same node and this PST2 cluster was more related to *stx2*- phages from highly virulent non-O157 strains, such as *E. coli* O104:H4 2011C-3493 [41], O103:H2 [42], and O103:H25 [43] when compared to PSTs 1 or 3.

**Fig. 5:**
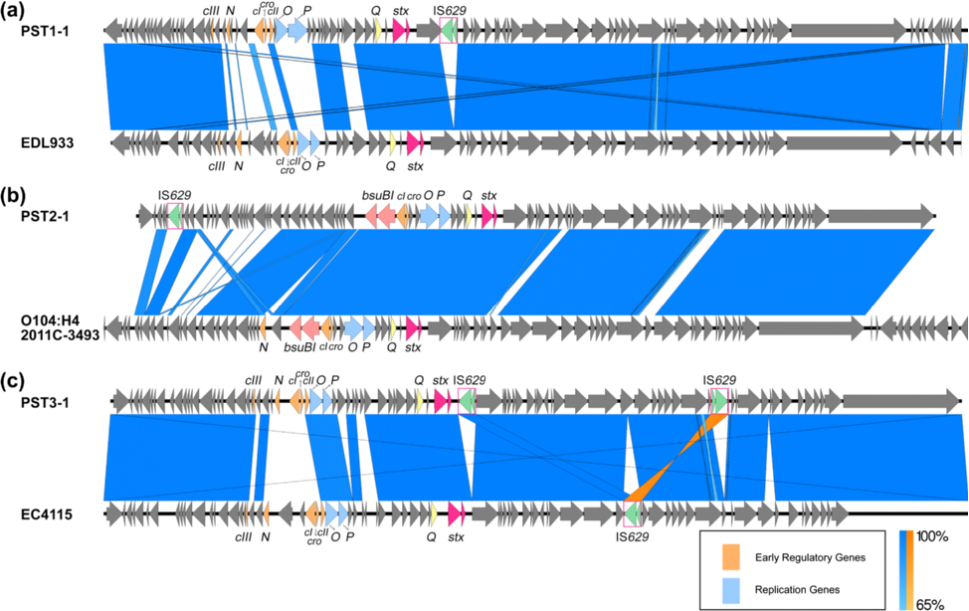
Alignment of PST1-1, PST2-1, and PST3-1 to related phage from *E. coli* O157:H7 strains Sakai, O104:H4 2011C-3493, and EC4115, respectively. BLAST comparisons of (**a**) PST1-1 and phage from EDL933, (**b**) PST2-1 and phage from O104:H4 2011C-3493 and (**c**) PST3-1 and phage from EC4115 were visualized using Easyfig [77]. Blue shaded regions connect homologous sequences and orange shaded regions connect reverse complemented homologous sequences. Arrows represent ORFs. Light orange, light blue and light yellow arrows represent early regulatory genes, replication genes and antiterminator *Q*, respectively. Light pink arrows represent *bsuBI* restriction/modification system. Dark pink and green arrows designate the location of *stx2* and IS*629*, respectively

BLAST comparisons [34] were performed between representative PSTs from each of the 3 clusters and the nearest neighbor from Fig. 4. PST1-1 and the EDL933 *stx2a*- phage [19], differed mainly within the predicted phages’ early regulatory and replication region (Fig. 5a, Additional file 8: Table S7). For PST2-1 and the phage in strain *E. coli* O104:H4 2011C-3493, phage regulatory and replication regions showed a higher degree of similarity (Fig. 5b, Additional file 8: Table S7). Noteworthy, the *bsuBI* restriction/modification system in PST2-1 is also present in the O104:H4 2011C-3493 phage genome [41]. Most differences between the two phages were found within regions encoding ORFs with no assigned functions. The most evident difference between PST3-1 and the EC4115 phage was within a region encoding genes for *N*, *cI*, and *cro* (Fig. 5c, 78).

### *E. coli* O157:H7 host genome affects Stx2 production

**Fig. 6:**
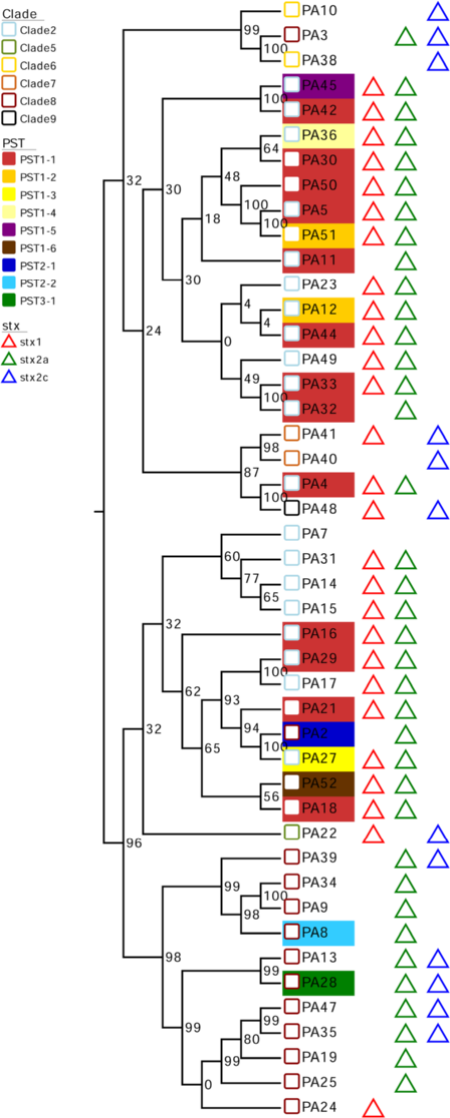
O157:H7 whole genome phylogeny and *stx2a*-converting phage PST comparison suggests multiple acquisitions of PST2 phages. Whole bacteria genomes of 45 O157:H7 strains were aligned using Mugsy [44]. The phylogenetic tree was inferred using RaxML with 1000 bootstrap replicates [45], and visualized as a cladogram due to high variation in branch length. PST1, PST2, and PST3 clusters are shaded in yellow, light purple and orange respectively. Corresponding metadata were added with Evolview [79]

**Fig. 7:**
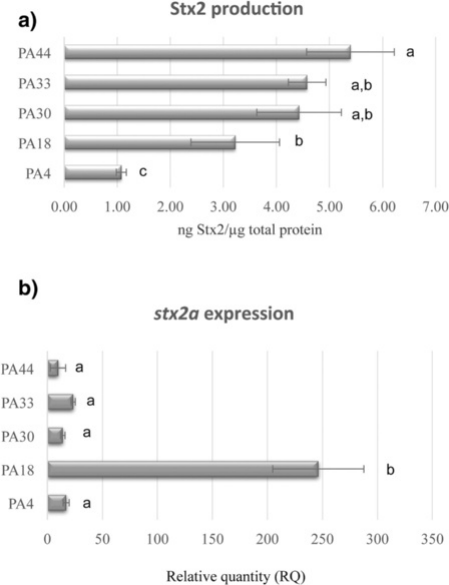
Host factors modulate *stx2a* expression levels. (**a**) Whole genomes of 14 O157:H7 strains with PST1-1 were aligned using Mugsy [44] and the phylogenetic tree was inferred using RaxML [45] with 100 bootstrap replicates and visualized using Figtree [88]. (**b)** Stx2 production was quantified by ELISA 2 h after ciprofloxacin induction. Bars labeled with different letters were significantly different (*P* < 0.05)

Our analyses identified for the first time strains of *E. coli* O157:H7 (PA4, PA18, PA30, PA33, and PA44) carrying *stx2a*-converting phages (PST1-1) that were 100 % identical on the phDNA level and had the same insertion site (*wrbA*). These strains also carry an *stx1*-converting phage [28]. This provided us with the unique opportunity to use non-manipulated strains to ask whether factors apart from the *stx2a*-converting phage genome impact toxin production. To first investigate the genetic relatedness of the host strains, we established a phylogenomic framework derived from whole genome alignments of shared regions, excluding prophage, for all 45 *E. coli* O157:H7 genomes [44, 45] (Fig. 6). Strains PA4, PA18, PA30, PA33 and PA44 are phylogenetically positioned on separate branches indicating that, as expected, no two chromosomes genomes were identical. Next, we measured the Stx2a levels and *stx2a* expression after ciprofloxacin phage-induction (Fig. 7a). The lowest Stx2a producer was PA4, at approximately 1 ng/μg of protein, and the highest producer was PA44, at approximately 5 ng/μg of protein. PA18 produced 3.2 ng/μg, which was significantly different from the previous two strains, while toxin production from strains PA30 (4.4 ng/μg) and PA33 (4.6 ng/μg) was only different from PA4. Surprisingly, *stx2a* transcript levels were similar for all strains except for PA18, which was greater than 10-fold above that measured with PA4, PA30, PA33, or PA44 (Fig. 7b). This observed disconnect indicates that qPCR does not always reflect the level of Stx2 secreted by *E. coli* O157:H7.

## Discussion

The progression and severity of HUS is thought to correlate with the amount of Stx2 produced by *E. coli* O157:H7 [46]. *E. coli* O157:H7 strains vary by their toxin production potential and this likely results in different virulence potentials [22, 47]. Molecular methods previously grouped *E. coli* O157:H7 strains into three lineages and nine clades [14, 48, 49]. Lineage I has been more frequently observed with isolates of human origin (at least in the United States) than were lineage I/II or lineage II isolates [48, 49]. This is presumably due, in part, to higher levels of Stx2 produced by lineage I isolates compared to other lineages [22]. As production of Stx2 is directly linked to phage replication [50], it is not surprising that we were most successful in isolating phDNA for sequencing from lineage I isolates. We only obtained sufficient DNA from 3 out of 12 clade 8 (lineage I/II) strains, which some reports suggested are high Stx2 producers [51], although another study [22] suggested Stx2 levels are at least one-log less than lineage I isolates. We were also unable to isolate sufficient phDNA from lineage II (clade 7) isolates (PA40, PA41) or from a clade 9 isolate (PA48); these groups of isolates are also typically low toxin producers [22]. While other temperate phages in *E. coli* O157:H7 are inducible [52], we only obtained sufficient DNA to close genomes for two others isolated from PA2 and PA8 that are related to a phage designated Sp7 in strain Sakai [30]. In contrast, the coverage for all *stx2a*-converting phages sequenced here was 117–573-fold. This data is consistent with the observations that most prophage in the *E. coli* O157:H7 genome are defective [52] and that the genome copy number of *stx2a*-converting phage upon mitomycin C induction is at least two orders of magnitude higher than for other prophages.

Previous studies suggested that genetic polymorphisms between *stx2a*-converting phage genomes could impact levels of toxin production [20, 23, 53]. Our study identified eight new genotypes of *stx2a*-converting phage, almost doubling the number of complete phage genomes varieties deposited. We observed that one major driving force of phage genome diversity is in IS*629* copy number and location variations. This is in accordance with previous studies that identified multiple copies of IS*629*, especially in the prophage, prophage-like and plasmid regions [24, 25, 54, 55]. This provides us with several testable hypotheses for how polymorphisms in the phage genome may impact toxin levels. For example, *stx2a* expression and subsequent release of toxin from the bacteria requires induction of the lytic cycle. Noteworthy, we observed a disruption of a putative *roi* in PST1-3 and PST1-6, which encodes a DNA-binding protein that inhibits the activation of the lytic cycle [56]. Therefore, we speculate that the specific IS*629* insertion in PST1-3 and PST1-6 may increase toxin production in strains carrying these phage sequence types. Once Stx2 is produced, efficient release of the toxin requires lysis of the bacteria. In PST1-5, the gene encoding a putative lysozyme was disrupted which could reduce the amount of toxin released. Inactivation of adenine DNA methyltransferase genes such as that observed in PST2-1 may additionally affect toxin production through de-repressing the lytic cycle [57].

The other major source of phage genome diversity, as noted before [19], was located within early regulatory regions. The diversity of this region is driven by homologous recombination between different lambdoid prophages [30, 58, 59]. Previous studies have also suggested that polymorphisms in early regulatory genes could lead to differential *stx* expression [23, 37, 38]. For example, the cooperative binding of CI dimers to operator sites designated O_L_ and O_R_ maintains the phage in its lysogenic state through repressing transcription from the P_L_ and P_R_ promoters, respectively. The copy number of each operator site can vary, for example *E. coli* O157:H7 strain EDL933 has two O_L_ sites surrounding P_L_ that are designated O_L1_ and O_L2_, while the strain Sakai also has an O_L3_ [37]_._ Four hours post antibiotic phage-induction, Stx2a production is greater for EDL933 than Sakai [60], and one reasonable hypothesis could be that this is caused by decreased repression at O_L_ [37, 38]. As with the PST1 phage from Sakai, our PST3 phage also had three identifiable O_L_ and O_R_ sites, however, we were unable to identify operator sequences or some early regulatory genes in the PST2 cluster phages. Whether one or more of these differences are responsible for the reduced quantities of toxin produced by strain PA2 compared to Sakai and EDL933 [60] remains to be determined.

Both PST2 and PST3 phages are found within clade 8 of *E. coli* O157:H7, which is a subgroup associated with high incidence of hospitalization and HUS [14, 15]. We found that PST2 phages were highly similar to the Stx2a phage of *E. coli* O104:H4 2011C-3493, a Stx2a-expressing enteroaggregative *E. coli* (EAEC) that was responsible for a severe outbreak in Germany in 2011 [41]. A related phage designated TL-2011C (Fig. 4) is found within the genome of an *E. coli* O103:H25 strain that caused a small outbreak in Norway [43] that again was characterized by a high HUS rate. Future research should investigate whether these phages are indicators of high Stx2a producing strains.

Diversity within the phage genome undoubtedly plays an important role in toxin production, however other factors encoded within the host may also influence the phage lifestyle [61]. Our assembly of phage genomes identified several *E. coli* O157:H7 strains carrying identical *stx2a*-converting prophages that were integrated at the same genomic site. Importantly, all of these strains also contain *stx1*-converting phage, which are known to decrease Stx2 production in *E. coli* O157:H7 strains carrying both phages [27]. Thus, by using wild-type clinical isolates that have been minimally passaged since isolation, we could test the hypothesis that genes external to the phage impact toxin production. Indeed, we observed a greater than five-fold variation in Stx2 production between PA4 and PA44, demonstrating that future studies investigating the mechanism(s) behind differences in Stx expression must take both phage and chromosomal host background into consideration. We also noted some discordance between toxin protein and transcript levels (Fig. 7), which is similar to observations with *E. coli* O157:H7 strain TW08612 [51]. This suggests that caution must be taken when assessing potential virulence of *E. coli* O157:H7 by qPCR alone. The identification of highly similar phage was also observed with *stx2*-converting phage from *E. coli* O104:H4 isolates [62], highlighting a remarkable stability contrasted to the mosaic structure that also exists between phage as reported here and elsewhere [63].

Although a single acquisition of the *stx2a*-converting phage was suggested in the well-established stepwise evolution model from O55:H7 to O157:H7 [10, 11, 13], prophage regions are also known as hotspots of horizontal gene transfer. Laing *et al*. [40] used both complete genome and WGS to compare *stx*-converting phage phylogeny with their host bacterial phylogeny and found that the two phylogenies generally agree with each other. However, the phage genomes extracted from WGS were not necessarily fully assembled and this may have influenced the accuracy of this analysis [40]. Unlike most whole genome shotgun sequencing studies, which do not allow for precise assembly of *stx*-converting phage regions, our study accomplished this by enriching for and sequencing phDNA after removal of bacterial genomic DNA. Our data suggest that PST1 phages in general evolved vertically along with the host. We did however observe that *E. coli* O157:H7 strains PA2 and PA8, both of which carry PST2 phages, occupy distinct nodes on the tree generated from whole genome alignments (Fig. 6). While it is possible that this demonstrates mobilization of the PST2 phages, our limited genomic data set is clearly insufficient to make any firm conclusions.

## Conclusion

This study expands our knowledge of the genetic diversity within *stx2a*-converting phages, and suggests that both phage and bacterial sequences, as well as phage insertion sites modulate Stx2 production. Sequencing additional *stx2a*-converting phages is justified, but should probably use other approaches as the strategy we took may bias results towards high phage producing strains. Next Generation Sequencing (NGS) long read technologies, such as PacBio, have been used by other investigators for accurate phage genome assemblies when other (related/homologous) phages are present as well [64]. As a larger number of complete genomes become available, a more comprehensive comparison to other Enterobacteria phage lambda will be possible that accounts for exchange of phage through genetic recombination, establishing whether the phage genotypes described could be considered distinct species. This study also serves as robust foundation for future work investigating whether the phage genome polymorphisms observed impact the virulence phenotype, particularly Stx2 production, both in pure culture and in combination with other members of the gut microbiota.

## Methods

### Bacterial strains and culture media

The 45 *E. coli* O157:H7 isolates used in this study were obtained from the Pennsylvania Department of Health and were originally characterized by Hartzell *et al*. (2011) [28]. All isolates were stored at −80 °C in 10 % glycerol and grown in Luria-Bertani (LB) [65] at 37 °C.

### Phage DNA isolation and sequencing

Phage DNA (phDNA) was isolated as previously described [66]. Briefly, each of the 45 isolates (Additional file 1: Table S1) was grown overnight in LB at 37 °C with constant shaking. To induce the phages’ lytic cycle, each overnight culture was diluted in LB supplemented with 45 ng/ml ciprofloxacin to an optical density of 0.05 at 600 nm (OD_600_) and incubated for 8 h at 37 °C with shaking. Supernatants were collected after centrifugation (4000 × *g*, 10 min, 4 °C) and filtration (0.2-μm cellulose acetate filter; VWR, Philadelphia, PA) and treated with 0.6 Unit/ml DNase I (Promega, Madison, WI) and 1.8 μg/ml RNase (Promega, Madison, WI) at 37 °C for 1 h to digest gDNA and RNA. The treated supernatants were incubated o/n with 10 % (wt/vol) polyethylene glycol 8000 (Promega, Madison, WI) and 1 M NaCl at 4 °C. After centrifugation (5000 × *g*, 2 h, 4 °C), each pellet was resuspended in SM buffer (0.58 % NaCl, 0.2 % MgSO_4_ · 7H_2_O, 1 M Tris-Cl [pH 7.5], and 0.01 % gelatin) and treated with 50 μg/ml proteinase K (EMD, Darmstadt, Germany) at 65 °C for 1 h to digest the phage capsid protein and release phDNA. A phenol-chloroform (1:1 vol/vol) extraction was performed to remove protein and the Clean and Concentrator kit (Zymo Research, Irvine, CA) was used to further purify and concentrate phDNA. The double-strand DNA (dsDNA) was quantified using the dsDNA BR assay kit (Qubit, Grand Island, NY). Purified phDNA (>200 ng) was sequenced using a Roche GS 454 FLX+ system (454 Life Science, Branford, CT) at the Penn State Genome Core Facility. Sequencing of gDNA was performed by the University of Maryland Institute for Genome Sciences on the Illumina HiSeq 2000.

### Phage genome assembly

The phDNA reads for each of the 22 isolates were assembled *de novo* using SeqMan NGen (DNASTAR, Madison, WI). The average sequencing depth ranged from 117 to 573. For each isolate, BLAST analysis of contigs (>1000 bp) was performed against the non-redundant nucleotide database in GenBank to identify the best matching phage genome to use as a reference for scaffold building. Reference genomes were VT2Phi (accession HQ424691) [40, 67] for strains PA2 and PA8, *E. coli* O157:H7 EC4115 (accession CP001164) [53] for strain PA28, and Sakai (accession BA000007) [30] for the remaining phage. Gaps were closed by manual curation, and the draft genome was corrected for homopolymers using Illumina gDNA reads previously generated from the *E. coli* O157:H7 host strain (Additional file 1: Table S1). Finally, PCR followed by DNA sequencing was used to validate the location of the IS elements within the phages.

### Phage genome annotation

Phage genomes were auto-annotated using RAST [68] and PHAST [69]. The GenBank files generated by RAST were then manually curated in Artemis [70] using the following principles: (i) prophage integration sites and tRNA genes carried by the phage were identified by PHAST, if present; (ii) open reading frames (ORFs) predicted to encode proteins of 39–94 amino acids, when present on the opposite DNA strand from and overlapping a predicted operon, were deleted if no known function had been ascribed to that ORF; (iii) if RAST and PHAST identified a different start codon for the same ORF, the Glimmer-based [71] prediction of PHAST was followed; (iv) ORFs identified by RAST but not PHAST were kept if one of the following was observed: a BLAST match was identified with a gene of known function, a Pfam motif was identified [72], Psortb [73] identified at least one secondary structure feature, or Fugue [74] returned a match with confidence interval greater than 99 % ; (v) for ORFs identified by PHAST but not RAST, guideline iv was followed except the Fugue confidence interval was reduced to greater than 95 %. A few exceptions to these rules exist: (a) in PA2, PA8 and PA28, the 3’ end of the integrase overlaps with the 3’ end of the downstream ORF, however both ORFs were annotated; (b) in PA45, ORF49 and 50 are predicted to be convergently transcribed and overlap at their stop codons. We kept these ORFs since guideline iv and v supported each.

### Comparative genome analysis

Illumina HighSeq reads were mapped to assemblies with CLC Genomics Workbench 7.04 (CLC Bio) to assess quality and coverage of predicted SNPs. All SNPs had a coverage above 100 × and Phred scores of 64. Phage genome alignments were generated with progressive Mauve [75] and visualized with Geneious R7.1.7. [76]. Comparisons of gene and protein sequences were performed using ClustalW packaged in MegAlign (DNASTAR Inc, Madison, WI) to identify SNPs and calculate sequence percent identities. BLASTN alignments of phage genomes were visualized using EasyFig [34, 77].

### Phylogenetic trees

Multiple sequence alignments were performed via Mugsy for whole genome alignments of both *E. coli* chromosomal and phage genomes [44]. The alignment was then imported into Galaxy [78] to convert the MAF alignment file into a fasta file containing a single entry per strain. The maximum likelihood phylogenetic trees were inferred using RAxML V7.2 [45], with the following parameters: rapid bootstrapping and GTRGAMMA with 100 and 1000 bootstraps for chromosomes and phages, respectively. Trees and strain-associated metadata were visualized with Evolview [79].

The phage genome tree that includes phages from GenBank was generated using the method and parameters described by Laing et al. [40]. Panseq [39] was used to cut phage genomes into 500 bp fragments. The “core genome” was defined by combining fragments present in ≥ 3 phage genomes at > 85 % identity. Other parameters were ‘nucB’ = ‘200’, ‘nucC’ = ‘50’, ‘nucD’ = ‘0.12’, ‘nucG’ = ‘100’ and ‘nucL’ = ‘11’. The presence or absence of core genome fragments in each phage genome was used for clustering. Uncorrected P-distance was used to build a neighbor-joining tree by SplitsTree 4 [80].

### BLAST Score Ratio Analysis

The phage proteome for each of the 9 phages sequence types were predicted using the Prodigal [81] gene prediction software. The concatenated predicted ORFs were then de-replicated with Usearch [82] and run through comparative BLAST score ratio (BSR) analysis [31] using TBLASTN [34]. For each of the predicted proteins, a BLASTP raw score was obtained for the alignment against itself (REF_SCORE) and the most similar protein (QUE_SCORE) in each of the genomes. Dividing the QUE_SCORE obtained for each query genome protein by the REF_SCORE normalized these scores and constitutes the BLAST score ratio (BSR). Proteins with a normalized BSR of <0.4 were considered to be non-homologous. A normalized BSR of 0.4 correspond to two proteins being 30 % identical over their entire length [31, 83].

### Stx2 gene expression and toxin production

Stx2 gene expression and toxin production was quantified using reverse transcription-qPCR and ELISA, respectively. Briefly, overnight LB cultures of each strain were diluted to an OD_600_ of 0.05 with fresh LB media supplemented with 40 ng/mL of ciprofloxacin [84] and incubated for 2 h at 37 °C with shaking. For reverse transcription-qPCR, total RNA was isolated using the Ambion PureLink mini kit (Life Technologies, Grand Island, NY). The RNA quantity and quality was assessed at absorbance of 260 nm using UV-spectrophotometer. RNA was treated with amplification grade DNase I (Invitrogen, Grand Island, NY) and used for cDNA synthesis with the Masterscript kit (5 PRIME, Gaithersburg, MD). The primer pairs for *stx2a* (5’-TCCCGTCAACCTTCACTGTA-3’ and 5’-GCGGTTTTATTTGCATTAGC-3’, target subunit B gene) [85] and *tufA* (5’-TGGTTGATGACGAAGAGCTG-3’ and 5’- GCTCTGGTTCCGGAATGTAA-3’) [86] were used in conjunction with Go-Taq**®** qPCR Master Mix (Promega, Madison, WI) and MicroAmp Fast optical real-time 96-well PCR plates (Applied Biosystems, Grand Island, NY) in an ABI StepOne PLUS Real-Time PCR system (Applied Biosystems, Grand Island, NY). Dissociation curves were analyzed for all reactions to verify single peaks/products. Expression levels of *stx2a* were normalized against *tufA* using ABI StepOne PLUS System SDS software (Applied Biosystems, Grand Island, NY) and shown in relative quantity (RQ). Three biological replication were conducted for each strain. For ELISA analysis, pre-warmed polymyxin B (6 mg/mL) was added to the induced culture and incubate for 10 min at 37 °C to lyse all the bacteria. Supernatants were collected after centrifugation (4000 × g, 10 min, 25 °C) and diluted to measurable concentrations. The 96 well polystyrene microtiter strip plates (Thermo Scientific, Waltham, MA) coated with 2.5 μg of ceramide trihexoside (Matreya Biosciences, Pleasant Gap, PA) were used for ELISA. After coating, the plates were incubated o/n at 4 °C with blocking buffer (4 % bovine serum albumin in 0.01 M phosphate buffer saline with 0.05 % Tween20). Samples (100 μl) were added to each well and incubated for 1 h at room temperature with shaking. After removing the samples, the wells were washed with PBS-Tween (PBST) buffer for five times. Mouse anti-Stx2 (Santa Cruz Biotech, Santa Cruz CA) (1 μg/ml, 100 μl) was added to each well. Plates were incubated for 1 h at room temperature with shaking, followed by five PBST washes. Goat anti-mouse IgG peroxidase conjugated (0.1 μg/mL, 100 μl) secondary antibody was added and incubated for 1 h at room temperature with shaking. After five PBST washes, 100 μl of 1-Step Ultra TMB (3,3’,5,5’ tetramethylbenzidine) was added to each well, and the plates were incubated for 10 min at room temperature followed by the addition of an equal volume of stopping solution (2 M H_2_SO_4_). The OD at 450 nm was examined using a spectrophotometer. A standard curve was constructed for each plate and three technical repeats were performed for each sample. Total protein for each sample was measure using the Bradford assay. Three biological replicates were conducted for each strain. Data were analyzed using one-way analysis of variance (ANOVA) and Tukey’s test by R Version 3.0.2.

## Additional files

Additional file 1: Table S1. Information on *Escherichia coli* O157:H7 strains used in this study, and Genbank accession numbers for whole genome shotgun sequences and for completed phage genomes. (PDF 88 kb) Additional file 2: Figure S1. Phage genomes were aligned with Mugsy and visualized in Geneious. Conserved regions compared to *E. coli* O157:H7 strain PA4 are highlighted in gray. Mean pairwise identity over all columns represents 100 % in dark green, 30–99 % in green-brown and below 30 % in red. (PDF 84 kb) Additional file 3: Table S2. SNPs observed within the PST1 cluster of phage. (PDF 59 kb) Additional file 4: Table S3. Copy number and insertion site of IS*629* elements in phage genomes sequenced in this study. (PDF 56 kb) Additional file 5: Table S4. Sequence identity of selected genes encoded within the 3 PSTs defined in this study. (PDF 62 kb) Additional file 6: Table S5. Putative operator sequences identified in PST1 and PST3 phage. (PDF 45 kb) Additional file 7: Table S6. GenBank information and insertion site of previously sequenced *stx2*-converting phage used in this study [89–93]. (PDF 71 kb) Additional file 8: Table S7. Sequence percent identity of regulatory genes and proteins, comparing each PST to the closest related previously sequenced phage. (PDF 59 kb)
