# Supplementary material for: Escherichia coli O157:H7 strains harbor at least three distinct sequence types of Shiga toxin 2a-converting phages
Source: BMC Genomics. 2015 Sep 29;16:733. doi: 10.1186/s12864-015-1934-1 (PMC4587872; doi:10.1186/s12864-015-1934-1)
Supplement: Additional file 1: Table S1. — Information on Escherichia coli O157:H7 strains used in this study, and Genbank accession numbers for whole genome shotgun sequences and for completed phage genomes. (PDF 88 kb) [file 12864_2015_1934_MOESM1_ESM.pdf]

Table S1. PA strain information

Strain Name[a]	Clade[b]	*stx* Allele(s)	Genome accession[d]	Phage accession[e]
PA2	8	*stx2a*	AOEL00000000	KP682371
PA3	8	*stx2a, stx2c*	AKLC01000000	
PA4	2.9	*stx1a, stx2a*	AMUA01000000	KP682372
PA5	2.9	*stx1a, stx2a*	AKLD01000000	KP682373
PA7	2.9		AMTQ00000000	
PA8	8	*stx2a*	AOEO00000000	KP682374
PA9	8	*stx2a*	AKLE01000000	
PA10	6	*stx2c*	AKLF01000000	
PA11	2.9	*stx2a*	AOEI00000000	KP682375
PA12	2.9	*stx1a, stx2a*	*This study*	KP682376
PA13	8	*stx2a, stx2c*	AOEK00000000	
PA14	2.9	*stx1a, stx2a*	AKLG01000000	
PA15	2.9	*stx1a, stx2a*	AKLH01000000	
PA16	2.9	*stx1a, stx2a*	*This study*	KP682377
PA17	2.9	*stx1a, stx2a*	*This study*	
PA18	*	*stx1a, stx2a*	*This study*	KP682378
PA19	8	*stx2a*	AOEJ00000000	
PA21	*	*stx1a, stx2a*	*This study*	KP682379
PA22	5.2	*stx1a, stx2c*	AKLI00000000	
PA23	2.9	*stx1a, stx2a*	AMUB0100000	
PA24	8	*stx1a*	AKLJ01000000	
PA25	8	*stx2a*	AKLK01000000	
PA27	2.9	*stx1a, stx2a*	*This study*	KP682380
PA28	8	*stx2a, stx2c*	AKLL01000000	KP682381
PA29	2.9	*stx1a, stx2a*	*This study*	KP682382
PA30	*	*stx1a, stx2a*	*This study*	KP682383
PA31	2.9	*stx1a, stx2a*	AKLM01000000	
PA32	2.9	*stx2a*	AKLN01000000	KP682384
PA33	2.9	*stx1a, stx2a*[c]	AKLO01000000	KP682385
PA34	8	*stx2a*	AMTR01000000	
PA35	8	*stx2a, stx2c*	AOES00000000	
PA36	2.9	*stx1a, stx2a*	*This study*	KP682386
PA38	6	*stx2c*	AMUO01000000	
PA39	8	*stx2a, stx2c*	AKLP01000000	
PA40	7	*stx2c*	AKLQ00000000	
PA41	7	*stx1a, stx2c*	AKLR01000000	
PA42	2.4	*stx1a, stx2a*	AKLS01000000	KP682387
PA44	2.9	*stx1a, stx2a*	*This study*	KP682388
PA45	2.4	*stx1a, stx2a*	AMUD01000000	KP682389
PA47	8	*stx2a, stx2c*	AOEM00000000	
PA48	9	*stx1a, stx2c*	AOEN01000100	
PA49	2.9	*stx1a, stx2a*	AMUC01000000	
PA50	*	*stx1a, stx2a*	*This study*	KP682390

PA51	*	*stx1a, stx2a*	*This study*	KP682391
PA52	*	*stx1a, stx2a*	*This study*	KP682392

[a] For all 45 isolates, the date and geographic location of isolation, PFGE pattern and LSPA type were previously reported by Hartzell *et al*. (2011).

[b] Clade types were determined *in silico* or reported previously (Hartzell *et al*., 2011). * Unable to assign a clade because of missing SNP positions due to sequence quality.

[c] Strain PA33 was previously reported as an "*stx2* variant" (Hartzell *et al*. 2011). However, the phage genome sequence reported here identified it as *stx2a.*

[d] For isolates previously sequenced, GenBank accession numbers are reported. The 13 isolates sequenced during this study were submitted to the Short Read Archive under Bioproject PRJNA274145.

[e] GenBank accession numbers for phage sequenced in this study.
