# Supplementary figures and images for: Escherichia coli O157:H7 strains harbor at least three distinct sequence types of Shiga toxin 2a-converting phages
Source: BMC Genomics. 2015 Sep 29;16:733. doi: 10.1186/s12864-015-1934-1 (PMC4587872; doi:10.1186/s12864-015-1934-1)

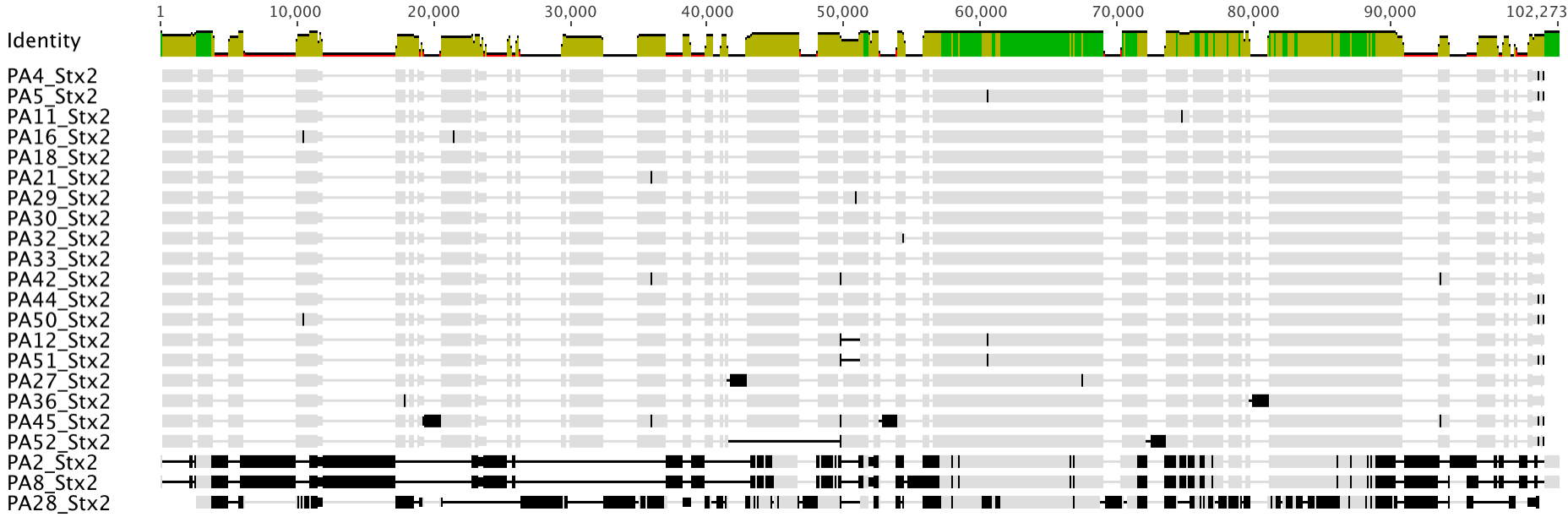

Supplement: Additional file 2: Figure S1. — Phage genomes were aligned with Mugsy and visualized in Geneious. Conserved regions compared to E. coli O157:H7 strain PA4 are highlighted in gray. Mean pairwise identity over all columns represents 100 % in dark green, 30–99 % in green-brown and below 30 % in red. (PDF 84 kb) [file 12864_2015_1934_MOESM2_ESM.pdf]
