# Supplementary material for: Escherichia coli O157:H7 strains harbor at least three distinct sequence types of Shiga toxin 2a-converting phages
Source: BMC Genomics. 2015 Sep 29;16:733. doi: 10.1186/s12864-015-1934-1 (PMC4587872; doi:10.1186/s12864-015-1934-1)
Supplement: Additional file 3: Table S2. — SNPs observed within the PST1 cluster of phage. (PDF 59 kb) [file 12864_2015_1934_MOESM3_ESM.pdf]

Table S2. SNPs observed comparing phage within the PST1-1 cluster

Position on phage genome	Host Strain	SNPs using PA4 as reference	Predicted function	Amino acid change
5453	PA50, PA16	G-A	intergenetic	-
9377	PA16	T-G	Predicted operator O_{R2}	-
16107	PA42, PA21	A-G	hypothetical	K-R
24363	PA42	G-T	hypothetical	synonymous
25476	PA29	G-A	IS*629*	P-S
27482	PA32	A-G	intergenetic	-
32341	PA5	C-T	portal protein	R-W
43963	PA11	C-T	tail fiber	T-I
58904	PA42	A-C	hypothetical	synonymous
