# Supplementary material for: Escherichia coli O157:H7 strains harbor at least three distinct sequence types of Shiga toxin 2a-converting phages
Source: BMC Genomics. 2015 Sep 29;16:733. doi: 10.1186/s12864-015-1934-1 (PMC4587872; doi:10.1186/s12864-015-1934-1)
Supplement: Additional file 4: Table S3. — Copy number and insertion site of IS629 elements in phage genomes sequenced in this study. (PDF 56 kb) [file 12864_2015_1934_MOESM4_ESM.pdf]

Table S3. IS*629* insertion site

**PST Type**	**Copy of IS*629***	**Gene Disrupted**
PST1-1	1	phage protein
PST1-3	2	putative DNA-binding protein Roi
		same as PST1-1
PST1-4	2	same as PST1-1
		hypothetical protein
PST1-5	3	inter-genetic region
		same as PST1-1
		phage lysozyme,
PST1-6	3	putative DNA-binding protein Roi
		same as PST1-1
		hypothetical protein
PST2-1	1	adenine DNA methyltransferase
PST2-2	1	inter-genetic region
