# Supplementary material for: Escherichia coli O157:H7 strains harbor at least three distinct sequence types of Shiga toxin 2a-converting phages
Source: BMC Genomics. 2015 Sep 29;16:733. doi: 10.1186/s12864-015-1934-1 (PMC4587872; doi:10.1186/s12864-015-1934-1)
Supplement: Additional file 5: Table S4. — Sequence identity of selected genes encoded within the 3 PSTs defined in this study. (PDF 62 kb) [file 12864_2015_1934_MOESM5_ESM.pdf]

Table S4. Sequence identity of selected genes from the 3 PST clusters

***cIII* (DNA)**	**PST1**	**PST3**
**PST1**	-	98.18
**PST3**	-	-

***cIII* (Amino Acid)**	**PST1**	**PST3**
**PST1**	-	98.15
**PST3**	-	-

***N* (DNA)**	**PST1**	**PST3**
**PST1**	-	50.58
**PST3**	-	-

***N* (Amino Acid)**	**PST1**	**PST3**
**PST1**	-	19.32
**PST3**	-	-

***cI*(DNA)**	**PST1**	**PST2**	**PST3**
**PST1**	-	62.81	48.72
**PST2**	-	-	48.57
**PST3**	-		-

***cI* (Amino Acid)**	**PST1**	**PST2**	**PST3**
**PST1**	-	49.77	14.08
**PST2**	-	-	17.59
**PST3**	-	-	-

***cro* (DNA)**	**PST1**	**PST2**	**PST3**
**PST1**	-	48.85	46.70
**PST2**	-	-	56.70
**PST3**	-	-	-

***cro* (Amino Acid)**	**PST1**	**PST2**	**PST3**
**PST1**	-	9.84	18.18
**PST2**	-	-	17.74
**PST3**	-	-	-

***cII* (DNA)**	**PST1**	**PST3**
**PST1**	-	97.31
**PST3**	-	-

cII (Amino Acid)	PST1	PST3
**PST1**	-	94.9
**PST3**	-	-

O (DNA)	PST1	PST2	PST3
**PST1**	-	50.92	57.03
**PST2**	-	-	61.98
**PST3**	-	-	-

O (Amino Acid)	PST1	PST2	PST3
**PST1**	-	12.71	8.81
**PST2**	-	-	16.03
**PST3**	-	-	-

P (DNA)	PST1	PST2	PST3
**PST1**	-	49.94	70.33
**PST2**	-	-	56.74
**PST3**	-	-	-

P (Amino Acid)	PST1	PST2	PST3
**PST1**	-	7.12	11.59
**PST2**	-	-	13.36
**PST3**	-	-	-

Q (DNA)	PST1	PST2	PST3
**PST1**	-	97.70	100.00
**PST2**	-	-	97.70
**PST3**	-	-	-

Q (Amino Acid)	PST1	PST2	PST3
**PST1**	-	98.61	100.00
**PST2**	-	-	98.61
**PST3**	-	-	-

Lysozyme (DNA)	PST1	PST2	PST3
**PST1**	-	91.57	93.26
**PST2**	-	-	92.32
**PST3**	-	-	-

Lysozyme (Amino Acid)	PST1	PST2	PST3

**PST1**	-	97.74	96.05
**PST2**	-	-	94.92
**PST3**	-	-	-

**Tail fiber (DNA)**	**PST1**	**PST2**	**PST3**
**PST1**	-	100.00	100.00
**PST2**	-	-	100.00
**PST3**	-	-	-

**Tail fiber (Amino Acid)**	**PST1**	**PST2**	**PST3**
**PST1**	-	100.00	100.00
**PST2**	-	-	100.00
**PST3**	-	-	-
