# Supplementary material for: Escherichia coli O157:H7 strains harbor at least three distinct sequence types of Shiga toxin 2a-converting phages
Source: BMC Genomics. 2015 Sep 29;16:733. doi: 10.1186/s12864-015-1934-1 (PMC4587872; doi:10.1186/s12864-015-1934-1)
Supplement: Additional file 6: Table S5. — Putative operator sequences identified in PST1 and PST3 phage. (PDF 45 kb) [file 12864_2015_1934_MOESM6_ESM.pdf]

Table S5. Putative operator sequence of PST1 and PST3

	**PST1**[a]	**PST3**
$\mathbf{O_{R1}}$	TACAACGCTTTGTATTT	ATAAATGACTGAT
$\mathbf{O_{R2}}$	CAATACAATTTGTTCTA	ATAACTATATTTAT
$\mathbf{O_{R3}}$	TAGTACGATATGTACTT	ATAACCTAAGTTAT
$\mathbf{O_{L1}}$	TATTACGCTCTGTACTG	ATAACCACGGTTAT
$\mathbf{O_{L2}}$	ATGCACAATGTGTATTT	ACAACAATTGTTAT
$\mathbf{O_{L3}}$	AAGTACATATTGTATTT	ATAACTCATGTTAT

[a] same as operator sequences in *E. coli* O157:H7 Sakai [37]
