# Supplementary material for: Escherichia coli O157:H7 strains harbor at least three distinct sequence types of Shiga toxin 2a-converting phages
Source: BMC Genomics. 2015 Sep 29;16:733. doi: 10.1186/s12864-015-1934-1 (PMC4587872; doi:10.1186/s12864-015-1934-1)
Supplement: Additional file 7: Table S6. — GenBank information and insertion site of previously sequenced stx2-converting phage used in this study [89–93]. (PDF 71 kb) [file 12864_2015_1934_MOESM7_ESM.pdf]

Table S6. GenBank information for *stx2*-converting phage

Host Strain or Phage Name[a]	Accession number	Insertion site[a]	Genomic region	Reference
Sakai	BA000007	*wrbA*	1246012-1308719	[30]
EDL933	AE005174.2	*wrbA*	1330829-1392498	[58]
Xuzhou21	CP001925.1	*wrbA*	1247053-1310658	[89]
O103:H2 12009	AP010958.1	tRNA(*argW*)	2861734-2924345	[42]
O111:H- 11128	AP010960.1	*yecE*	2392300-2440434	[42]
EC4115	CP001164.1	tRNA(*argW*)	3234765-3297047	[53]
TW14359	CP001368.1	tRNA(*argW*)	3189636-3251918	[90]
O104:H4 2011C-3493	CP003289.1	*wrbA*	3248372-3317120	[41]
O104:H4 2009EL-2071	CP003301.1	*wrbA*	3298273-3366761	[41]
O104:H4 2009EL-2050	CP003297.1	*wrbA*	3240628-3309116	[41]
Stx2-86	AB255436	-	-	-
Stx2 phi I	AP004402	-	-	[91]
Stx2 phi II	AP005154	-	-	[92]
Min27	EU311208	-	-	[93]
TL-2011C	JQ011318	-	-	[43]
vB_EcoP_24B	HM208303	-	-	[20]
VT2Phi	HQ424691.1	-	-	[40]

[a] Insertion sites are only reported when phages were sequenced as part of a full genome project
