# Supplementary material for: Escherichia coli O157:H7 strains harbor at least three distinct sequence types of Shiga toxin 2a-converting phages
Source: BMC Genomics. 2015 Sep 29;16:733. doi: 10.1186/s12864-015-1934-1 (PMC4587872; doi:10.1186/s12864-015-1934-1)
Supplement: Additional file 8: Table S7. — Sequence percent identity of regulatory genes and proteins, comparing each PST to the closest related previously sequenced phage. (PDF 59 kb) [file 12864_2015_1934_MOESM8_ESM.pdf]

Table S7. Sequence percent identity of regulatory genes and proteins, comparing each PST to the closest related previously sequenced phage.

***cIII* (DNA)**

**PST1 and EDL933**	98.79
**PST3 and EC4115**	97.58

***cIII* (Amino Acid)**

**PST1 and EDL933**	98.15
**PST3 and EC4115**	98.15

***N* (DNA)**

**PST1 and EDL933**	71.43
**PST3 and EC4115**	47.92

***N* (Amino Acid)**

**PST1 and EDL933**	58.89
**PST3 and EC4115**	17.76

***cI* (DNA)**

**PST1 and EDL933**	49.56
**PST2 and O104:H4 2011C-3493**	100.00
**PST3 and EC4115**	59.59

***cI* (Amino Acid)**

**PST1 and EDL933**	18.75
**PST2 and O104:H4 2011C-3493**	100.00
**PST3 and EC4115**	14.62

***cro* (DNA)**

**PST1 and EDL933**	18.84
**PST2 and O104:H4 2011C-3493**	100.00
**PST3 and EC4115**	26.47

***cro* (Amino Acid)**

**PST1 and EDL933**	18.75
**PST2 and O104:H4 2011C-3493**	100.00
**PST3 and EC4115**	14.62

***cII* (DNA)**

**PST1 and EDL933**	95.96

**PST3 and EC4115**	88.55

***cII* (Amino Acid)**	
**PST1 and EDL933**	95.92
**PST3 and EC4115**	86.73

***O* (DNA)**	
**PST1 and EDL933**	57.03
**PST2 and O104:H4 2011C-3493**	95.73
**PST3 and EC4115**	98.62

***O* (Amino Acid)**	
**PST1 and EDL933**	8.81
**PST2 and O104:H4 2011C-3493**	98.22
**PST3 and EC4115**	99.68

***P* (DNA)**	
**PST1 and EDL933**	70.76
**PST2 and O104:H4 2011C-3493**	97.22
**PST3 and EC4115**	96.72

***P* (Amino Acid)**	
**PST1 and EDL933**	10.73
**PST2 and O104:H4 2011C-3493**	96.90
**PST3 and EC4115**	97.85

***Q* (DNA)**	
**PST1 and EDL933**	100.00
**PST2 and O104:H4 2011C-3493**	95.63
**PST3 and EC4115**	100.00

***Q* (Amino Acid)**	
**PST1 and EDL933**	100.00
**PST2 and O104:H4 2011C-3493**	95.83
**PST3 and EC4115**	100.00
